# Distancing Bonus Or Downscaling Loss? The Changing Livelihood of Us Online Workers in Times of COVID‐19

**DOI:** 10.1111/tesg.12455

**Published:** 2020-06-28

**Authors:** Fabian Stephany, Michael Dunn, Steven Sawyer, Vili Lehdonvirta

**Affiliations:** ^1^ Oxford Internet Institute University of Oxford 1 St Giles OX1 3JS Oxford UK; ^2^ Department of Management & Business Skidmore College 815 North Broadway Saratoga Springs NY 12866 USA; ^3^ School of Information Studies Syracuse University 343 Hinds Hall Syracuse NY 13244‐1190 USA

**Keywords:** Online labour markets, multi‐method study, freelance work, COVID‐19, telework

## Abstract

We draw on data from the Online Labour Index and interviews with freelancers in the United States securing work on online platforms, to illuminate effects of the COVID‐19 pandemic. The pandemic's global economic upheaval is shuttering shops and offices. Those able to do so are now working remotely from their homes. They join workers who have always been working remotely: freelancers who earn some or all of their income from projects secured via online labour platforms. Data allow us to sketch a first picture of how the initial months of the COVID‐19 pandemic have affected the livelihoods of online freelancers. The data shows online labour demand falling rapidly in early March 2020, but with an equally rapid recovery. We also find significant differences between countries and occupations. Data from interviews make clear jobs are increasingly scarce even as more people are creating profiles and seeking freelance work online.

## Graphical abstract

We draw on data from the Online Labour Index and interviews with freelancers in the United States securing work on online platforms, to illuminate effects of the COVID‐19 pandemic. Data allow us to sketch a first picture of how the initial month of the COVID‐19 pandemic has affected the livelihoods of online freelancers. The data show online labour demand falling rapidly in early March 2020, but with an equally rapid recovery. Interviews make clear jobs are increasingly scarce even as more people are creating profiles and seeking freelance work online.

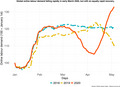

## Introduction

We combine data from the Online Labour Index (OLI) and an interview‐based panel study of freelance workers in the United States to provide insight into the changes in online labour markets relative to the ongoing pandemic arising from the global spread of the novel coronavirus, COVID‐19. We do so to contribute evidence and insight to the ways in which a global pandemic appears in the localised context of one of the economies most impacted. The freelance workers that are the focus of this analysis are those taking on projects and contracts for knowledge work: technology support, software development, bookkeeping, accounting, web content, writing and editing, and other types of cognitive work. The growth in online labour markets, and the project‐ or task‐based structure of freelance labour, provides a unique window into effects of a pandemic on work. The OLI provides a global perspective; the panel study provides for a more localised set of insights.

We pursue this work recognising that as the scope and breadth of the global COVID‐19 pandemic continues to grow, the implications to workers and labour markets grow more profound. Even conservative estimates of a contained global outbreak are showing significant global macroeconomic impacts (McKibbin & Fernando 2020). Similarly, models at the country level are showing serious economic impacts (Atkeson 2020). Early research is also showing distinct demographic and industry differences in the impact of the pandemic (Wenham *et al*. 2020; Stephany *et al*. 2020).

Building on the emerging COVID‐19 related research, we examine a specific subset of labour markets – the online labour market and its workers – to understand the near‐term impacts of the pandemic. Online freelancers are in precarious work arrangements generally, and the pandemic presents a particularly challenging scenario to them for at least three reasons. First, the online nature of their work makes them susceptible to greater competition (Dunn 2017). Second, freelance work is project‐based: there is little to no commitment between employer and worker beyond the specifics of the project's contract (Wood *et al*. 2019). Finally, in many countries their status as independent contractors leaves online freelancers in vulnerable positions especially during economic downturns. For example, in the United States, benefits such as health care are tied to formal, full‐time employment and are not provided to freelancers. Indeed, in comparison with many industrialised countries, there are relatively fewer labour and employment regulations governing non‐standard work arrangements in the United States (ILO 2016; McKay *et al*. 2019).

On the one hand, online labour markets could be experiencing a boost in demand as companies move operations online. On the other hand, the sharp economic downturn could be causing companies to reduce the use of online labour platforms alongside other types of non‐standard work. We address this issue by examining recent changes in the global demand for online labour. And while viruses may be blind to nation‐states, policies and interventions are nation‐state specific. Cross‐national comparisons can therefore provide insight on the economic implications of specific policies and interventions. Research has already begun to understand the economic implications of country specific interventions (e.g. Thunstrom *et al*. 2020). In this initial rapid analysis, we therefore use quantitative data to examine changes in the demand for online labour in three important regional economies with different countermeasures towards the pandemic: United States, Germany, and South Korea. The data show distinctly different geographic patterns between countries, with US data showing a particularly acute drop in demand for workers, with a simultaneously sharp increase in supply of available workers.

The data also make clear that not all occupations in the United States are experiencing the shocks similarly. Tech and software development occupations show a significant increase in both online labour demand and number of registered profiles. Because of the significant market shock evident in the United States and its notable occupational differences, we complement the quantitative view with interviews with US‐based online freelance workers, helping us to understand the significance, implications and lived experiences of the market shock to online freelance workers.

## Background

Online labour platforms are websites that mediate between buyers and sellers of remotely deliverable cognitive work (Horton 2010). The clients range from individuals and early‐stage startups to Fortune 500 companies (Corporaal & Lehdonvirta 2017). The sellers are either self‐employed independent contractors, or people in regular employment who earn additional income by moonlighting as freelancers via the Internet. The platforms match clients and workers using a variety of mechanisms, such as allowing clients to post projects for bidding, and allowing freelancers to post resumes for clients to evaluate. Besides matching, the platforms also handle contracting, time tracking, monitoring, billing, and dispute resolution, allowing the entire relationship to be carried out remotely.

Online labour platforms can be further subdivided into freelancing platforms (e.g. Upwork, TopTal, Fiverr) where payment is on an hourly or milestone basis, and microtask platforms (e.g. Amazon Mechanical Turk) where payment is on a piece rate basis (Lehdonvirta 2018). Of these, freelancing platforms appear to be much larger in terms of user numbers (Kässi & Lehdonvirta 2018). Online labour platforms are also sometimes called online gig platforms, but they are conceptually distinct from local gig economy platforms such as Uber or Deliveroo, which involve physical on‐site service delivery (Wood *et al*. 2019). The global market for online labour has grown approximately 50 per cent over the past three years (Kässi & Lehdonvirta 2018). But as the COVID‐19 pandemic is hitting the world's economies, causing a massive rise in unemployment in the United States, it is pertinent to ask how the pandemic is affecting the market for online labour.

### Pandemic's potential effects on online labour demand

There are several potential mechanisms through which the pandemic could be causing a positive demand shock for online labour. The pandemic appears to be forcing companies in affected countries to shift from collocated office work towards home‐based remote working arrangements, known in previous literature as telework (Huws *et al*. 1990) or telecommuting (Mokhtarian, 1991), and now colloquially referred to as ‘work from home’. The pandemic is also likely pushing companies to increase the use of virtual collaboration as a substitute to travel and face‐to‐face meetings. Telework and virtual collaboration have been slowly and unevenly gaining in popularity already since their introduction in the early 1990s. Now, there appears to be an unprecedented surge of interest towards them. For instance, interest in remote working and related search terms in Google search approximately tripled from its pre‐pandemic baseline to March 2020 (Clement 2020). The stock market value of teleconferencing software company Zoom Video Communications approximately doubled in the same time period.^1^

Given this surge of interest in remote work and virtual collabouration, it is conceivable that remote‐by‐design online labour markets could be seeing a significant demand boost. Companies looking to engage new contractors might now favour remote online contractors hired through web‐based platforms over on‐site contractors hired through conventional staffing agencies. Already before the pandemic, many skilled workers located in rural areas of the United States appear to have successfully used online labour markets to remotely access opportunities in urban areas (Braesemann *et al*. 2020). It is also possible that some companies might be moving existing contractor relationships to online labour platforms, in what is known as the ‘bring your own freelancer’ model (Corporaal & Lehdonvirta 2017). Platforms provide features for monitoring and managing contractors remotely, which can partly substitute for the missing in‐person controls (Braesemann *et al*. 2020). Short‐term contractors are typically subject to more performance‐ and outcome‐based controls than regular employees, whose loyalty may be sought with cultural and incentive‐based controls (Ouchi 1979).

Furthermore, the pandemic and the switch to remote work and virtual collaboration might also be creating additional demand for certain types of labour, some of which is supplied through online labour platforms. In particular, it is conceivable that there is a significant positive demand shock for information technology contractors who can help companies set up and maintain remote work and virtual collaboration infrastructure, and provide training on their use. Systems integration work and database management work might also increase as organisational reliance on systems over in‐person interactions increases. Large firms often have existing IT services outsourcing providers, but small‐ and medium‐sized companies may be turning to online labour platforms for these needs (Bunyaratavej *et al*. 2011).

However, it is also conceivable that the pandemic could be causing a significant reduction in demand for online labour, through a few possible mechanisms. Online labour is part of the broader category of non‐standard work, which includes other independent contractors and temporary workers. Many companies in the United States use non‐standard workers as a flexible buffer that can be rapidly reduced in economic downturns to protect core workers (Kalleberg 2003). Online labour can also be seen as a form of outsourcing (Lehdonvirta *et al*. 2019), which is likewise something that companies can adjust to respond to changing demand. Given that the pandemic and its public policy responses have in many countries already resulted in a general economic downturn of historic proportions, it is conceivable that companies could be cutting their use of online labour platforms to engage contractors. Already before the pandemic it was apparent that demand in the online labour market was very responsive to events such as public holidays (Kässi & Lehdonvirta 2018), and workers felt that demand for their services could fluctuate a lot (Lehdonvirta 2018).

Two opposing effects of the COVID‐19 pandemic on demand for online freelance labour are thus conceivable. On the one hand, to the extent that online labour is a substitute to on‐site labour, we can expect to see an increase in demand. The technical and organisational effort involved in switching to remote operations might also cause an increase in the demand for specific types of online labour, especially IT services. We will refer to all these demand‐increasing mechanisms collectively as the *distancing bonus*. On the other hand, to the extent that online labour is a complement to general economic activity, we can expect to see a decrease in demand, as companies facing declining revenues reduce non‐essential spending, including external online contractors. We refer to this as the *downscaling loss*.

### Pandemic's potential effects on online labour supply

The pandemic can also be expected to have a significant impact on the supply of labour on online labour markets. The supply generally speaking consists of two margins: the number of workers offering their services through online platforms, and the number of hours that they are willing to supply (Horton 2010). The number of workers offering services online might conceivably increase, because the pandemic and its countermeasures have led to record‐high unemployment in many countries, freeing up skilled workers (del Rio‐Chanona *et al*. 2020). Workers who were already offering a small number of hours online on top of their regular work (i.e. moonlighting; Pesole *et al*. 2018) might increase the hours offered as a result of being laid off. On the other hand, workers who are not laid off from their regular jobs might decrease the number of hours they supply through online labour markets, to signal loyalty to their employers. Both moonlighters as well as existing full‐time online freelancers might also decrease the hours supplied online as a result of falling ill or having increased care and housework duties, as schools and daycare centres close or family members fall ill.

### Geographic and temporal variation in the effects

The pandemic's possible positive and negative effects on online labour supply and demand are likely to vary across space and time, for a variety of reasons. Some reasons have to do with the dynamics of the pandemic itself. The pandemic unfolds in phases, from initial discovery to growing spread and eventual public and private countermeasures, followed by an easing up of the countermeasures and possible additional waves of infection (Bedford *et al*. 2020). Owing to differences in initial entry time, infection rates, and response speed, different countries are at different phases, with different impacts on economic activity. Country differences in public and private countermeasures, severity of the epidemic, and underlying economic conditions are also likely to generate geographic variation in effects seen in online labour markets (ILO 2020).

Moreover, dynamics of the online labour market itself provide plenty of reasons to expect geographic differences (Beerepoot & Lambregts 2015). Generally speaking, a clear Global North‐‐Global South trade pattern is evident in online labour markets, where the majority of employers are located in high‐income countries (Kässi & Lehdonvirta 2018) while most workers are located in low‐ and middle‐income countries (Lehdonvirta et al. 2019). Different employer countries have different demand profiles in terms of the occupations they are buying from online labour platforms, although these differences are surprisingly small (Kässi & Lehdonvirta 2018).

Changes in supply and demand in different countries are ultimately reflected in the global aggregate online labour supply and demand. Although there is evidence of preference and discrimination in online labour markets (Galperin & Greppi 2019), overall the market functions globally, with workers with similar skills and experience considered reasonably good substitutes for each other regardless of location (Lehdonvirta *et al*. 2019).

The resulting overall effects on the market have substantive policy implications. If and when online labour demand experiences a distancing bonus effect while supply remains relatively constant, then the growing online labour market could offer an avenue for laid‐off workers to recover some of their earnings. But if the downscaling loss effect dominates and/or the market is flooded with additional labour supply, then people thus far earning their main income through online labour platforms are likely to join the ranks of self‐employed people urgently in need of financial assistance. In the following section we present a rapid initial quantitative assessment of which effects dominate, across time and selected countries.

## Quantitative Evidence from the Online Labour Index

We draw on the OLI for a first quantitative assessment of how online labour markets are affected by the COVID‐19 pandemic. The OLI is an index that measures the utilisation of online labour platforms over time and across countries and occupations (Kässi & Lehdonvirta 2018). It serves a similar function as conventional labour market statistics on new vacancies. The index is constructed by continuously collecting data on tasks and projects posted on major online labour platforms in near real‐time. The results of the OLI are published as an open data set and an interactive online visualisation, updated daily (http://ilabour.oii.ox.ac.uk/online‐labour‐index/). In this rapid initial assessment, we examine the effects of the pandemic on the aggregate global market, followed by three important regional economies. Finally, we present additional analyses pertaining to the United States.

**Figure 1:**
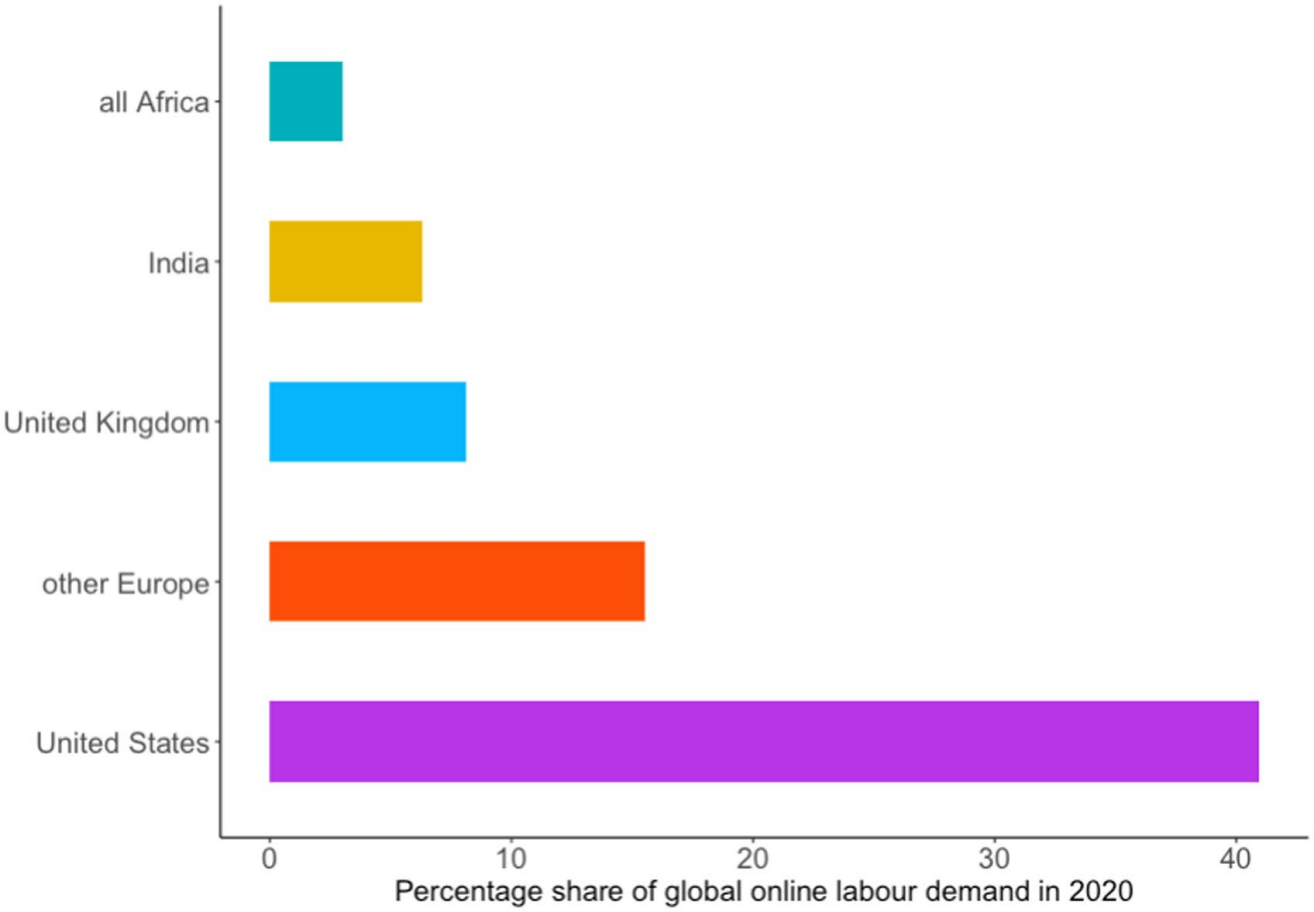
Globally, in 2020, the largest share of online labour demand stems from the United States. [Colour figure can be viewed at wileyonlinelibrary.com]

As discussed in the literature review, online labour markets have a distinct geography. Our data allows us to provide a detailed picture of how the demand for online labour is geographically distributed on the platforms monitored by the OLI. As shown in Figure 1, the largest share of online labour demand originates from employers based in the United States, who posted 41 per cent of all projects recorded in 2020. The second largest buyer country for online freelance work is the United Kingdom (8%), followed by India (6%). Europe excluding the United Kingdom generates 16 per cent of online labour demand, with Germany as the biggest demander in this group. Only three per cent of the demand for online workers comes from the entirety of Africa. Given these very uneven geographies of online labour demand, it is fair to assume that global developments on online labour markets are often driven by buyers from the United States.

### Changes in online labour demand

**Figure 2:**
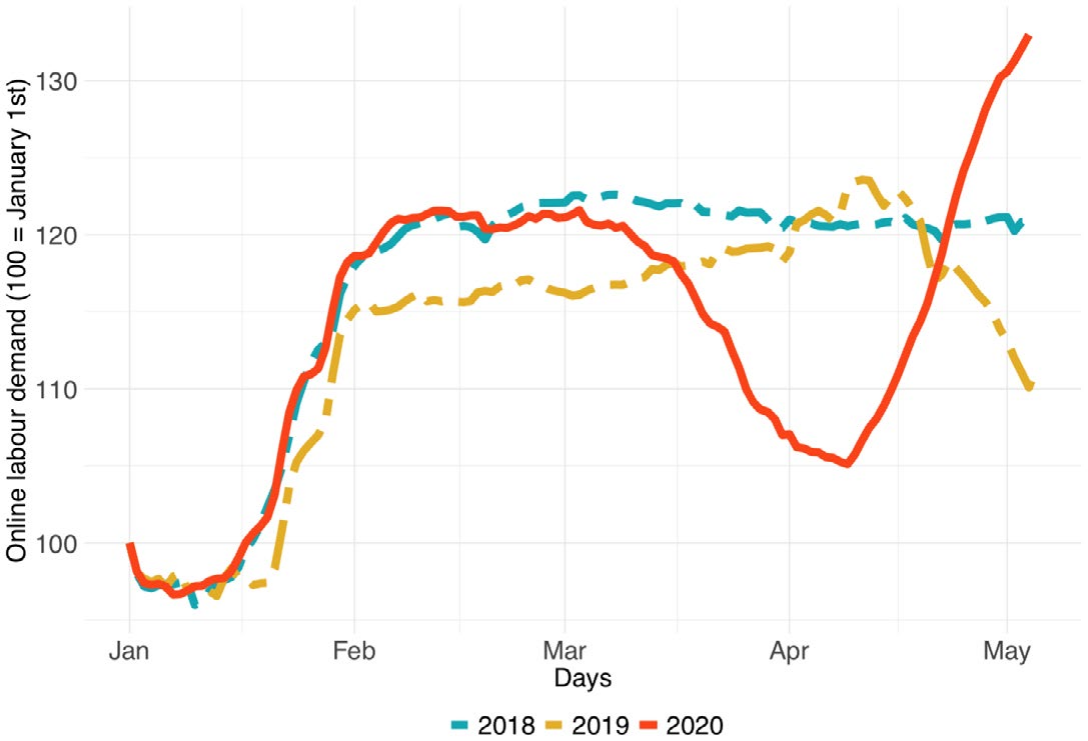
Demand on major online labour platforms, 1 January to 18 April (2018–2020), relative to the start of the year [Colour figure can be viewed at wileyonlinelibrary.com]

Over the past several years, the OLI has shown a clear seasonal pattern: demand drops during the year‐end holiday season, and then rises again to reach a plateau in February, which normally persists until May. However, as Figure 2 shows, this is not the case in 2020. By mid‐March, when the World Health Organisation declared that COVID‐19 had become a pandemic, the OLI was in deep decline, in comparison to 2018 and 2019. This finding indicates that the downscaling loss effect may be dominating over the distancing bonus effect. However, in early April 2020 the OLI began to rise again, surpassing by far the usual level of previous years by the end of April. This observation, on the other hand, indicates that at this stage of the pandemic, the distancing bonus may have started to dominate over the downscaling loss.

To further examine this fast‐changing dynamic, we are interested in examining the biggest demander country, the United States. However, the United States was not the first economy to be impacted by the pandemic and its countermeasures. It is useful to contrast it with other important regional economies with different COVID‐19 trajectories and responses. In this study, we limit ourselves to examining three important regional economies: Germany, South Korea, and the United States as the three economies confronted the pandemic at different points in time and opted for different countermeasures.

**Figure 3:**
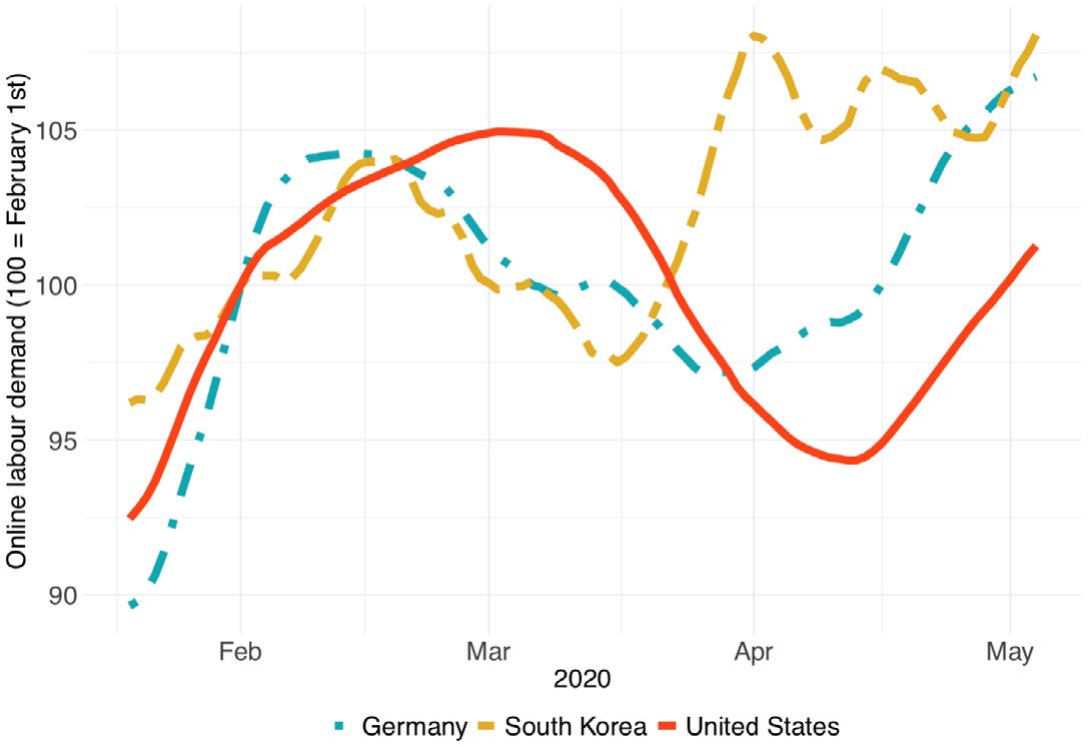
Online labour demand from the United States, Germany and South Korea, relative to the start of the year. [Colour figure can be viewed at wileyonlinelibrary.com]

Figure 3 shows a cross‐country comparison of online labour demand from Germany, South Korea and the United States. Each country shows a distinct pattern. South Korea was one of the first economies that had to face the consequences of COVID‐19 early in 2020. Demand from South Korea fell from mid‐February to mid‐March, but bounced back rapidly in late March. Demand from Germany similarly fell from February to March, and experienced a more modest rebound in April. Demand from the United States started falling roughly two weeks later than the demand from South Korea and Germany and fell furthest, but by early May was close to reaching pre‐crisis levels again.

These patterns are consistent with the idea that the pandemic reached the United States later than it reached South Korea and Germany. Moreover, in comparison to the United States, Germany and South Korea imposed countermeasures of different types and magnitudes. With the historical experience of the MERS outbreak in 2015, South Korea relied on mass testing and digital infection tracing, allowing the country to avoid a full lockdown (Normile 2020). Germany established early and localised testing, which allowed the government to impose only relatively moderate restrictions and to permit local business to open again in the beginning of April. Demand from the United States can also be seen bouncing back from mid‐April onwards, even though restrictions remained largely in place. This could reflect businesses adjusting to the new normal of remote work, rather than a return to pre‐pandemic operations.

Given the particularly clear drop in demand for online labour from the United States, we are interested in how this drop may vary across different types of work. The OLI categorises online labour into six different occupations: clerical and data entry, professional services, software development and technology, creative and multimedia, sales and marketing support, and writing and translation. For a more detailed description of these categories, see Kässi and Lehdonvirta (2018).

**Figure 4:**
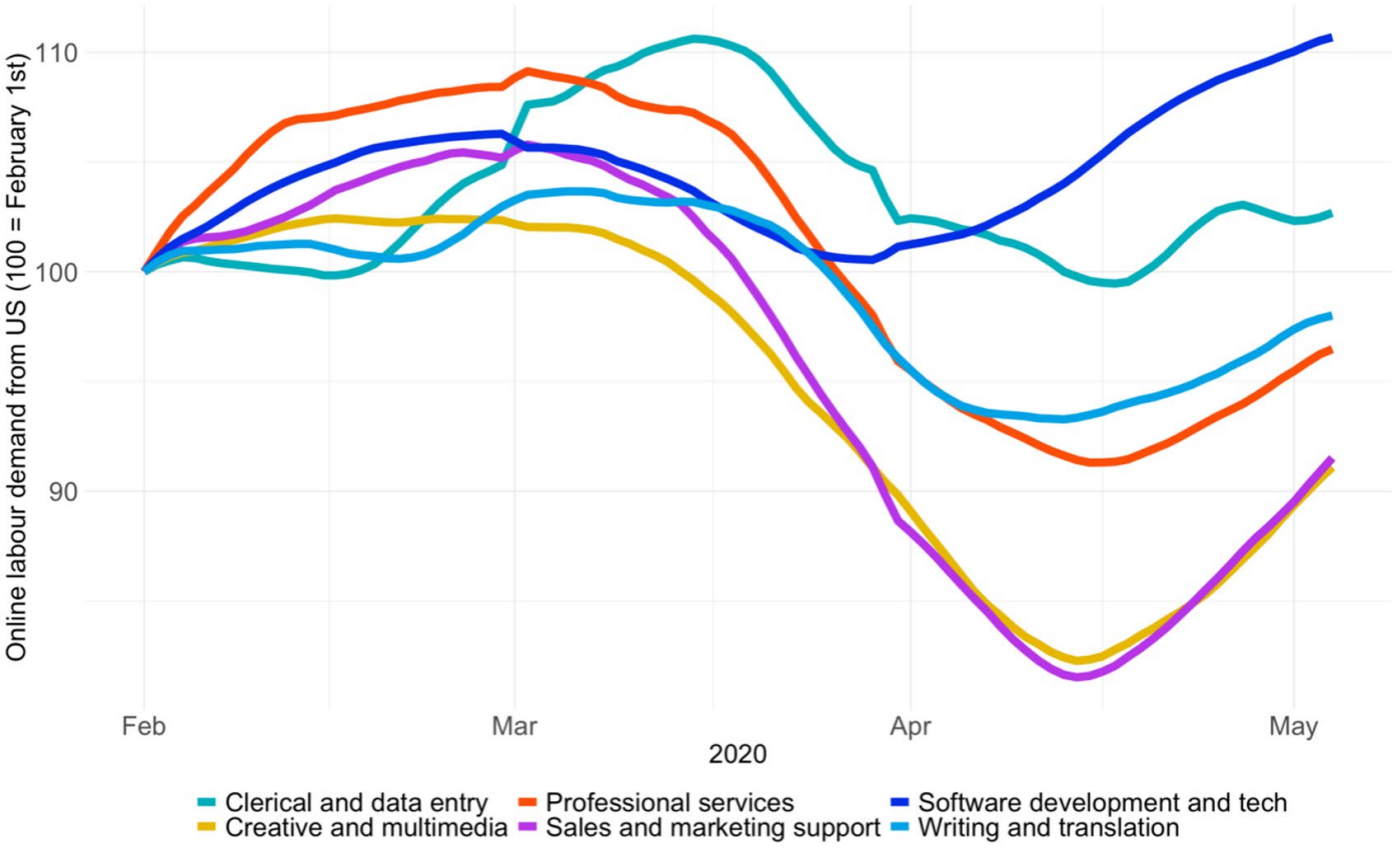
Online labour demand from the US, relative to the start of the year, by occupation

As Figure 4 shows, not all occupations have experienced a drop in demand. Demand in creative and multimedia or sales and marketing support has shrunk significantly as the pandemic has unfolded. But requests for projects in the software development and technology category remain largely unaffected. This finding is consistent with an interpretation that companies are cutting non‐essential freelance contracts, such as marketing and sales campaigns, while maintaining freelance outsourcing that is essential for continued business operations, such as tech support and database management. The figures are also consistent with the idea that the rapid push towards videoconferencing and other remote operations across companies has created additional demand for freelance IT specialists who are able to help with this.

### Changes in online labour supply [Colour figure can be viewed at wileyonlinelibrary.com]

The OLI does not provide us with a direct measure of labour supply, but we are able to observe the number of registered worker profiles on a smaller set of online labour platforms.^2^ This can be used as an imperfect proxy for the number of workers offering services through online labour platforms. We are not able to observe changes in the number of hours the workers are supplying.

**Figure 5:**
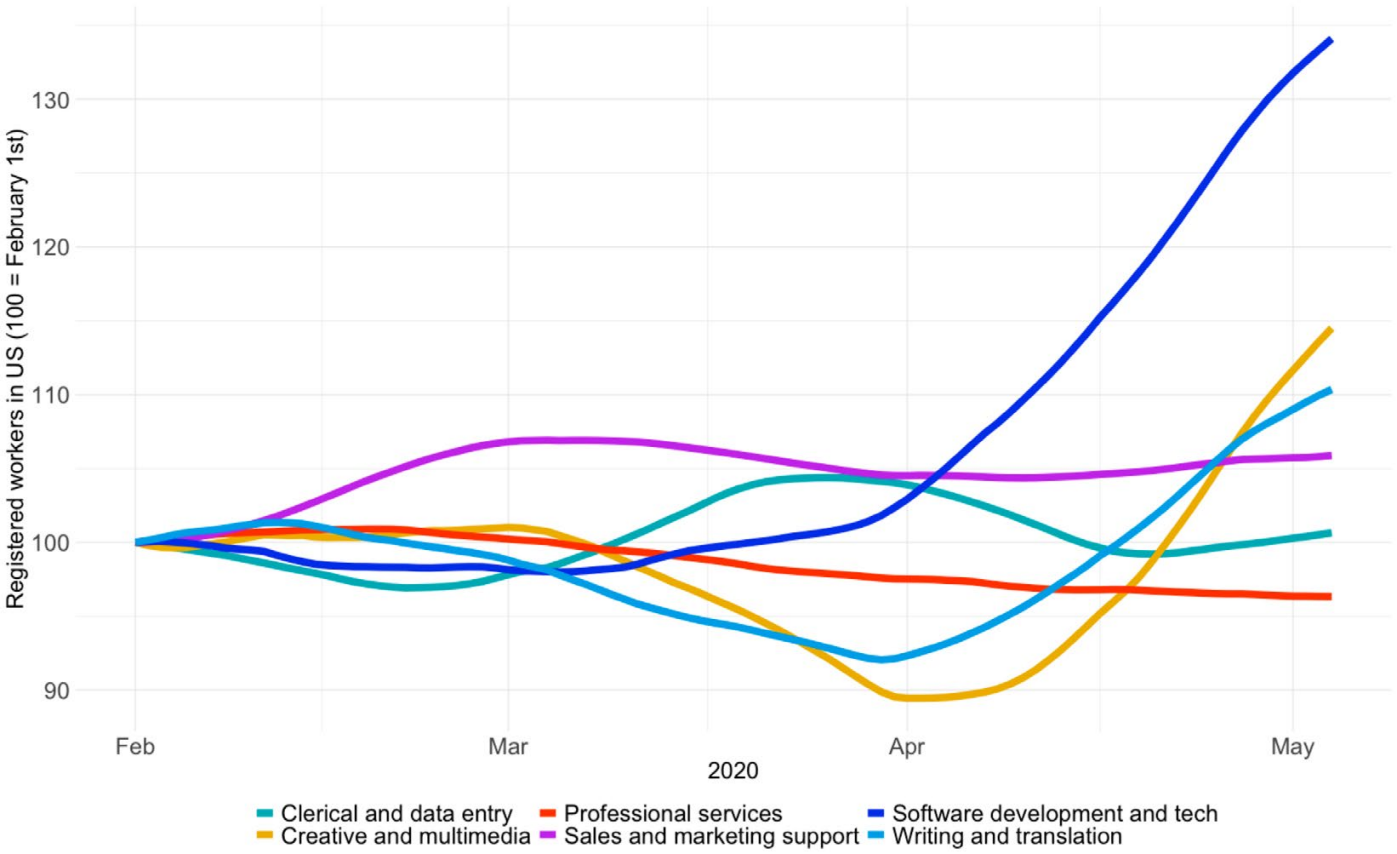
Registered online worker user accounts in the US, relative to the start of the year. [Colour figure can be viewed at wileyonlinelibrary.com]

An increase in the number of registered freelancer profiles in the United States is evident since the beginning of April. In particular, as shown in Figure 5, a very significant number of new freelancers have registered in the software development and technology category. Other occupations do not show a similar increase in registered workers. This is consistent with an interpretation that recently laid‐off workers across the economy are not registering en masse on online labour platforms to attempt to make up for lost income. Some workers are probably doing so, but to some extent the increased supply in the software development and technology category might also be attributable to the pull of increased opportunities due to growing demand.

In sum, while we observe a *downscaling loss* for most online labour occupations in the United States, software and tech jobs appear to profit from a *distancing bonus* effect. However, even in this category labour supply growth in the United States appears to have outpaced labour demand growth, suggesting that the workers are likely to be experiencing a tight market. To better understand the workers' experiences, we draw on data from an ongoing panel study in the United States that relies on structured interviews to provide insights on how these trends are being manifested through the lived experiences of workers.

## Insights From Interviews With Freelance Workers in the United States

Our interview data come from an ongoing panel study of 60 freelance workers who are located in the United States and seeking work online via the online labour platform Upwork (See http://upwork.com). Upwork is one of many online labour platforms and routinely seen as a dominant player. The focus on Upworkers serves as a window into the career plans and work strategies of freelance workers seeing work online. The study is designed around a carefully constructed sample of people who pursue freelance work as a primary or secondary source of income, and reflect a range of work types, skill levels, experience online, gender, ethnicity and success with this work.

Participants are hired and paid as they would for other jobs found on Upwork.^3^ Once hired, participants complete a 15' survey that provides us an overview of their working plans, outcomes and experiences and a 45' interview. The interview builds on the survey data and follows a carefully designed protocol of semi‐structured questions. Interviews are done by one of the six members of the digital work research group, a joint effort of Syracuse University and Skidmore College, both in New York state. The research team members were trained on the protocol and meet frequently and routinely to review the protocol, the data, and pursue interim analyses (as is customary in field studies).

Beginning in mid‐March 2020, we asked freelancers how they were faring in the face of the COVID‐19 pandemic. Since then, we have done 31 interviews. For this paper, we completed an interim analysis of these freelancers, reviewing the transcripts of the interview, drawing on the field notes, and looking to secondary sources for additional insight.^4^ Specific to comparing with the OLI, the panel study design and this initial analysis, relies on the job classifications provided by Upwork,^5^ grouped into three broader categories (See Table 1 for summary statistics):

1. Administrative work to include: accounting, customer service, translation, editing. In the OLI, this category roughly responds to the occupations ‘writing and translation’, ‘professional services’, and ‘clerical and data entry’.
2. Technology work to include: web, mobile and standard programming, engineering and architecture, data and analytics, IT and networking. In the OLI, this category roughly responds to the occupation ‘software development and tech’.
3. Creative work to include: design, graphics, sales and marketing, writing, and some one‐offs like crisis response public‐relations. In the OLI, this category roughly responds to the occupations ‘creative and multimedia’, ‘writing and translation’, and ‘sales and marketing support’.

**Table 1:** Summary statistics.

Job classification		Gender	
Administrative	32%	Female	61%
Technology	32%	Male	39%
Creative	36%		

		Hours a week spent looking for work	
Role of Freelance Work		Min	0
Primary Source of Employment	Additional Source of Employment	1st Qu.	1
53%	47%	Median	5
		Mean	11
		3rd Qu.	20
		Max	45

Age		Length on digital platforms (in years)	
Min	23	Min	0
1st Qu.	30	1st Qu.	2
Median	35	Median	3
Mean	37.99	Mean	5.25
3rd Qu.	45	3rd Qu.	8.50
Max	75	Max	20

Data from the panel study provide substantial evidence in support of downscaling loss effects. The freelancers with whom we spoke report that there are fewer jobs being posted. Freelancers who have long‐standing clients tell us these clients are pausing current projects and not adding new projects. Furthermore, freelancers indicate that there are many more people bidding for the jobs: sometimes six times more than even a month ago (e.g. 30+ bids now, versus five bids). This seems to align very well with the data presented in Figure 5. Freelancers are also reporting that in response to the increased number of people seeking work online, they are having to bid on more jobs to keep securing work, and often are bidding for work below their target salary rates, and even below minimum wage rates. While each freelancer's situation is unique, work‐seeking strategies in our initial analysis, seem to be externally focused. That is, while many have relied on existing relationships, their predictability of these relationships producing work are now more tenuous. In turn, freelancers indicated that they are diversifying and applying to different type of work, increasing the number of jobs they simultaneously are bidding for, bidding for lower paying contracts and lowering their hourly rates.

From this initial analysis we see little evidence of distancing bonus effects in the United States across the platforms broadly, although we see initial evidence of distancing bonus effects at the occupation level. At first these changes were not affecting one broad category of work more than another. By April 2020, those respondents in creative spaces (marketing, design, web content) noted work was slowing dramatically, while work supporting digital infrastructure (web services, back‐end web work) was steady or even increasing, corroborating with the data in Figure 4. This suggests that clients are starting to look into the concerning future and prioritising basic operations over customer‐facing efforts.

Beyond these insights on distancing bonus and downscaling loss effects, two additional observations from our interviews warrant mention. First, these US‐based freelancers are reporting changes in their life worlds that are reshaping their labour strategies and working arrangements. Nearly all of the respondents report spouses and partners being laid off or having to work from home, and that their children are now at home for schooling. These changes are requiring the freelancers to alter their own working arrangements and work availability – with most having less time to pursue work. The scope of these changes is increasing each week, and the changes are becoming more impactful as the economy continues to slow down. The current arrangements are no longer new, not yet normal, and uncertainty clouds things. These changes dampen the ability of workers to take on typical work loads, providing some opportunity for new entrants.

Finally, every one of the participants in the panel study is casting an increasingly worried eye on the longer term as the economic slowdown in the United States continues to press on most but the wealthy. Panel data make clear that fewer than 40 per cent of our respondents have health care: a profoundly disturbing finding given that many of their spouses and partners are losing jobs. Panel data also make clear that fewer than 40 per cent of our respondents had more than a few weeks of savings: seven weeks into massive social distancing and 22 million people losing their jobs, these workers are exhausting these reserves, well before the economic crisis is over. Taken together, the low number of freelancers with healthcare, and savings, showcases the precarity of the workspace and amplifies the implications of the changes to the online labour markets as demonstrated above.

## Discussion and Conclusion

Findings suggest the downscaling loss effect may be dominating over the distancing bonus effect. At the same time, the number of workers seeking income through online labour platforms is increasing. This, in turn, suggests freelancers earning their income through online labour platforms are more likely to see a tighter market and larger variations in their income in the face of a more uncertain online labour market. For the US‐based freelancers with whom we spoke, their situation is further complicated by the structural and legal landscape they face. To wit, their employment classification (as independent contractors) restricts access to social policies that could serve as a social safety net (e.g. access to healthcare and unemployment benefits). The broad and chaotic response by the United States' Federal Government^6^ in the face of the economic crisis is more poignant for freelance workers as their status relative to what counts as work often means they are not able to access unemployment benefits and may not be eligible for the stimulus checks associated with the CARES act. Given the social and labour policies, significant market shocks, like the pandemic, make visible the risks associated with non‐standard work arrangements in the United States.

These risks may be one of the reasons why the OLI figures for the United States vary so much more than do those of South Korea and Germany. While this analysis has focused on the United States, to fully understand the nuances and differences in the online labour market, future analysis exploring other countries is essential. Furthermore, the data also only provide a freelancer perspective, so understanding changes and behaviors of clients (employers) and their impact (and how they're impacted) would provide an important perspective in the market dynamics discussed here.

We see value in the further interrogation of the *downscaling loss* and *distancing bonus* effects from other analytic perspectives to include temporality, geography, and demography. The magnitude of downscaling and distancing are likely to change over time, as the pandemic and its consequences unfold. Moreover the effects are likely to vary across geography, due to national differences in economic structures, the phase and severity of the pandemic, and countermeasures adopted. We also see distinct differences in demands for different occupations. Considering the gendered and racial differences in occupations (e.g. women, for example, are overrepresented in the sales and creative occupations and minorities are overrepresented in service occupations, See https://contexts.org/blog/inequality‐during‐the‐coronavirus‐pandemic/#rea), we then may see certain groups disproportionately affected. As can be seen, one possible roadmap for near‐term research related to the pandemic can be: (i) the examination in the differences driven by occupation; (ii) analysis of the differences in country‐level trajectories; and (iii) further investigation of gender and racial disparities found in freelance work. These implications illustrate the questions that emerge from this early view into changes in the online labour markets for freelance work arising from the global pandemic.

At a broader level, the COVID‐19 pandemic produces a unique window of opportunity to gauge the interdependencies between the online labour market and the regular economy. Initially, the sharp decline in the online labour market closely mirrored the broader labour market. As the pandemic has continued, our analysis shows a rebound in the online labour market. This not only suggests that online labour markets can transcend its traditionally complementary role in the broader markets, but may play a key role in bridging gaps in the broader economy caused by sudden economic shocks. We speculate this is being manifested in at least three ways. First, firms are using more project‐based work, which is more conducive to the use of freelancers. Second, firms are increasingly becoming more accustomed to distributed and remote working arrangements, which is also more conducive to freelancers. While both of these are independent of the current pandemic, they are macro‐level changes in employment relations that we see as catalysts to the increases in the demand for online freelancers. Third, online platforms allow people to more easily seek freelance work, which blurs the boundaries between online and traditional labour markets. As a more efficient matching system for work, online labour markets help to alleviate the friction associated with job searches and allow workers to seek work beyond their local geography. All of these things allow online labour markets to normalise more quickly during periods of economic shock. As the evidence in our analysis suggests, the online labour market demand has recovered, and in some occupational areas has surpassed, relative to pre‐pandemic levels.

## Notes

1 See https://markets.businessinsider.com/news/stocks/zoomstock-price-surged-coronavirus-pandemic-video-work-from-home-2020-3-1029023594

2 The raw data (used in Figure 5) are collected by periodically sampling workers from four major online labour platforms: Fiverr, Freelancer, Guru, and PeoplePerHour – http://ilabour.oii.ox.ac.uk/measuring‐the‐supply‐of‐digital‐labour‐how‐the‐oli‐worker‐supplement‐is‐constructed

3 To reduce the pressure on having Upworkers bidding for work (which consumes precious bidding resources), they are hired directly: reaching out to invite them to take the work. About 6 of 10 do: acceptances and declinations are tracked. Research guidelines require paying those who agree to this job even if they do not finish: this work is voluntary and they can cease working at any time. About 1 in 10 begin, but do not finish. All who begin are rated five stars (as performance ratings matter greatly for online workers). Many of those who have completed the work leave a positive review of the work. There are no negative reviews.

4 For example, there are active sub‐threads for experiences with platform work, generally, and Upwork, specifically, to be found in the online platform Reddit, as well as Facebook groups devoted to both.

5 Upwork changes its categorisation of workers from time to time, making classification a challenge.

6 CARES Act, see: https://www.congress.gov/bill/116th-congress/senate-bill/3548/text?q=product+update
